# Standing on slopes – how current microprocessor-controlled prosthetic feet support transtibial and transfemoral amputees in an everyday task

**DOI:** 10.1186/s12984-017-0322-2

**Published:** 2017-11-16

**Authors:** Michael Ernst, Björn Altenburg, Malte Bellmann, Thomas Schmalz

**Affiliations:** Research Biomechanics, CR&S, Otto Bock HealthCare GmbH, Göttingen, Germany

**Keywords:** Amputee, Prosthetic, Standing, Microprocessor-controlled prosthetic feet, Prosthetic knee, Biomechanics, Strategies in standing

## Abstract

**Background:**

Conventional prosthetic feet like energy storage and return feet provide only a limited range of ankle motion compared to human ones. In order to overcome the poor rotational adaptability, prosthetic manufacturers developed different prosthetic feet with an additional rotational joint and implemented active control in different states. It was the aim of the study to investigate to what extent these commercially available microprocessor-controlled prosthetic feet support a natural posture while standing on inclines and which concept is most beneficial for lower limb amputees.

**Methods:**

Four unilateral transtibial and four unilateral transfemoral amputees participated in the study. Each of the subjects wore five different microprocessor-controlled prosthetic feet in addition to their everyday feet. The subjects were asked to stand on slopes of different inclinations (level ground, upward slope of 10°, and downward slope of −10°). Vertical ground reaction forces, joint torques and joint angles in the sagittal plane were measured for both legs separately for the different situations and compared to a non-amputee reference group.

**Results:**

Differences in the biomechanical parameters were observed between the different prosthetic feet and compared to the reference group for the investigated situations. They were most prominent while standing on a downward slope. For example, on the prosthetic side, the vertical ground reaction force is reduced by about 20%, and the torque about the knee acts to flex the joint for feet that are not capable of a full adaptation to the downward slope. In contrast, fully adaptable feet with an auto-adaptive dorsiflexion stop show no changes in vertical ground reaction forces and knee extending torques.

**Conclusions:**

A prosthetic foot that provides both, an auto-adaptive dorsiflexion stop and a sufficient range of motion for fully adapting to inclinations appears to be the key element in the prosthetic fitting for standing on inclinations in lower limb amputees. In such situations, this prosthetic concept appears superior to both, conventional feet with passive structures as well as feet that solely provide a sufficient range of motion. The results also indicate that both, transfemoral and transtibial amputees benefit from such a foot.

## Background

The loss of a lower limb is a dramatic incident in a person’s life. At least for now, a prosthetic replacement is the only possible solution to regain a normal life. The prosthetic replacement should enable and support daily life activities such as standing, walking, sitting down and standing up. For transfemoral amputees (TF), microprocessor-controlled prosthetic knee joints, which control stance and swing phase in walking, have been considered standard of care for two decades now and they continue to undergo further development. They allow the amputee to walk with a more physiological gait and loading pattern (e.g. [1–6]) and reduce the risk of falls (e.g. [5, 7–12]).

Contrary to prosthetic knee joints, lower limb amputees are not yet commonly fitted with microprocessor-controlled prosthetic feet (MPF). Nonetheless, the design of prosthetic feet is the subject of further development. The influence of foot stiffness [13], range of ankle motion [14, 15], hydraulic ankle damping [16, 17], timing and amount of push-off [18–20] as well as actively powered feet [21–23] are subjects of current research aiming to improve outcomes for lower limb amputees.

Conventional prosthetic feet like energy storage and return feet without an additional rotational joint allow only a limited range of ankle motion compared to human ones and can replace only partly the complex structure and function of the human foot (for range of motion in humans, see e.g. [24]). The limited adaptability in dorsi- and plantarflexion becomes evident while standing on uneven ground. It can be assumed to be lacking due to the absence of a rotational joint and the reduced loading of the spring compared to walking (about 50%).

The task of quiet standing is not problematic on level ground but might cause problems on inclines in terms of unnatural posture and challenging stability. The issue of stability and control in standing with a prosthetic foot was addressed in the past (for a review, see [25]) but, to our knowledge, not for standing on slopes of different inclinations simulating uneven ground.

To overcome the poor rotational adaptability, prosthetic manufacturers have developed different prosthetic feet with an additional rotational joint and implemented active control of resistance in dorsi- and plantarflexion and inclination-independent control of a dorsiflexion stop. It has not yet been sufficiently investigated to what extent these commercially available MPF support a natural posture while standing on inclines and which implemented function is most beneficial for lower limb amputees.

In the present study, we compared biomechanical outcomes for standing on slopes of different inclinations in lower limb amputees who were equipped with different prosthetic feet. In addition to their everyday foot, five microprocessor-controlled feet (MPF) of different manufacturers were tested. Different features were embedded in the MPF which should help amputees to stand in a more natural posture compared to commonly used energy storage and return feet without an additional rotational joint. The aim of the present study was to show which combination of features brings the most benefit for amputees as indicated by biomechanical parameters such as symmetry of ground reaction force, joint kinematics and kinetics and in comparison with non-amputees. We, therefore, compared the vertical ground reaction forces (vGRF) between sound and prosthetic side as well as the joint angles and torques of the legs for different situations. The hypothesis was that the design features embedded in the different MPF show distinct biomechanical outcomes when standing on inclines and that the level of amputation shows an influence as well.

## Method and setup

### Participants

Four unilateral transtibial amputees (TT: age 56.2 ± 12.0 years, mass 79.8 ± 8.1 kg, height 178 ± 4 cm, all male) and four unilateral transfemoral amputees (TF: age 44.5 ± 3.0 years, mass 81.9 ± 8.0 kg, height 178 ± 7 cm, all male) took part in this study. All had undergone amputation at least three years prior to enrollment, and all participants used a conventional, non-microprocessor-controlled foot in daily life. TF subjects used microprocessor-controlled knees in daily life (Table 1). The individual activity level classification of the subjects were K3 or K4 (for K-level classification, e.g. [26]). For reference, 20 able-bodied subjects (non-Amp: 22.5 ± 3 years, mass 72.7 ± 15 kg, height 178 ± 10 cm, 10 male and 10 female) were investigated.

**Table 1:** Subject data

Subject	Level of amputation	Age [years]	Weight [kg]	Height [cm]	Everyday prostheses
P#1	TT	48	68.5	183	Trias (Otto Bock)
P#2	TT	74	84.0	174	C-Walk (Otto Bock)
P#3	TT	48	79.5	177	Triton Harmony 1C62 (Otto Bock)
P#4	TT	56	87.0	178	C-Walk (Otto Bock)
Mean TT		56.5 ± 12.0	79.8 ± 8.1	178 ± 4	
P#5	TF	44	75.0	169	Triton 1C60 & Genium (Otto Bock)
P#6	TF	48	76.0	178	Triton 1C60 & C-Leg3 (Otto Bock)
P#7	TF	45	84.5	184	C-Walk & C-Leg3 (Otto Bock)
P#8	TF	41	92.0	182	Triton 1C60 & X3 (Otto Bock)
Mean TF		44.5 ± 3.0	81.9 ± 8.0	178 ± 7	
Mean all		50.5 ± 10.0	80.8 ± 7.5	178 ± 5	

The study was conducted in accordance with the Declaration of Helsinki and approved by the ethics committee of the University Medical Center Göttingen (UMG). Prior to participation, each subject was given a detailed explanation of the study, and written informed consent was obtained from all subjects.

### Prosthetic foot intervention

In this study five different microprocessor-controlled prosthetic feet (MPF) which were commercially available at the time of the study were used: Meridium (Otto Bock, Germany), Elan (Version 1; Blatchford, UK), Proprio (Össur, Iceland), Triton Smart Ankle (hereinafter referred as TSA) (Otto Bock, Germany), and Raize (Fillauer, USA). Furthermore, all TF subjects were equipped with the same microprocessor-controlled knee (Genium, Otto Bock, Germany) for the duration of the study. Note that TF subjects were not equipped with the Raize foot because it was not approved for this level of amputation. The everyday feet of the subjects were the only non-MPF investigated in this study. The MPF used offered different additional functions compared to the non-MPF (for details see Tables 2 and 3 and the manufacturers datasheets).

**Table 2:** Data of the used feet

	Everyday feet	Meridium	Elan	Proprio	TSA	Raize
ROM [plantarflexion, dorsiflexion]	(−,−)^a^	(22,14)°	(6,3)°^a^	(11,18)°^a^	(17,17)°^a^	(18,10)°^a^
mechanics	–	hydraulic	hydraulic	DC-Motor^b^	hydraulic	hydraulic
control	–	MP	MP	MP	MP	MP
manufacturer	Otto Bock (see Tab.1)	Otto Bock	Blatchford	Össur	Otto Bock	Fillauer

ROM in ° and used mechanics and control. ^a^additional ROM due to carbon spring, ^b^adjustable only during swing phase, *MP* microprocessor. Data is taken from manufacturer’s datasheets

**Table 3:** Featured functions of the feet related to walking and standing

Functions of the feet	
Everyday feet	No ankle joint, adaptations due to elastic deformation of the carbon spring only
Meridium	Real-time adaptation to inclines and declines in stance phase (i.e. ankle angle adaptation to terrain in the same step) during walking; dorsiflexion stop for standing after adaptation to the terrain (i.e. dorsiflexion is locked after reaching auto-adaptable relative home position); auto-adaptive heel height adaptation (different shoes, barefoot)
Elan	Free ankle joint hydraulically damped with limited ROM (see Table. 2) – situation detection of incline/decline/fast speed (adjusts plantar –and dorsiflexion resistances); additional adaptations due to elastic deformation of the carbon spring
Proprio	Incremental adaptation of the ankle angle to inclines and declines in swing phase via motor (i.e. adaptation process needs more than one step), locked joint in stance (i.e. no ankle rotation); additional adaptations due to elastic deformation of the carbon spring; manual heel height adaptation (different shoes, barefoot)
TSA	Incremental adaptation of the ankle angle to inclines and declines in stance phase; locked joint in stance; gait speed adaptation (enables 1° additional dorsiflexion for slow speed, 0.5° plantarflexion for fast walking); additional adaptations due to elastic deformation of the carbon spring; manual heel height adaptation
Raize	Incremental adaptation to inclines in stance phase; real-time adaptation to declines in stance phase; additional adaptations due to elastic deformation of the carbon spring; manual heel height adaptation

Featured functions of the feet which might influence the behavior of the subjects while standing and walking. Functions of the feet were derived from the manuals, user trainings and observations during patient fittings. Note that the MPF have additional functions (e.g. activity modes, standing up etc.) that are not described

In the first step, an optimized static and dynamic prosthetic alignment was ensured by a certified prosthetist. The amputees wore each foot at least one week to accommodate to it before the laboratory visit. Prior to the tests, the correct prosthetic setting was confirmed again. The order of tests started with the everyday feet and was different from subject to subject.

### Experimental setup and data acquisition

For the study, the subjects were asked to stand on slopes of different inclinations: standing on level ground (Level; 0° inclination), standing on an upward slope of 10° inclination (Up), and standing on a downward slope of −10° inclination (Down). The slopes were constructed with two wooden blocks (length 35 cm, width 10 cm with a 10° inclination. A slope of 10° was used to simulate a slope which is moderate for non-amputees but challenging for lower limb amputees (see also [5]). The blocks were covered with a thin layer of shoe sole material on both sides to increase the friction and to prevent slippage of either the block or the foot. Each block was placed on a separate force plate (Kistler 9287A, 1000 Hz, Kistler Group, Switzerland) at a distance that equaled the subjects normal stance width (usually about 15 cm between blocks; once determined individually, it was kept constant for all conditions). Kinematic data were recorded with 12 Vicon cameras (Vicon Bonita, 200 Hz, Vicon Motion Systems, UK). Markers were placed bilaterally on the toe (metatarsophalangeal joint or equivalent position), ankle (lateral malleolus or mechanical foot rotation axis), knee (compromise axis of rotation of the knee, vertical: ~2 cm above medial tibial plateau, horizontal: 60% anterior / 40% posterior [27], or mechanical axis of rotation), and trochanter major.

The subjects were introduced and trained to standing on the slopes with both feet. They chose a posture which would allow them to stand quietly for the recording period. Besides this, there were no recommendations given (e.g. regarding their posture).

The subjects stood in front of the slopes on level ground and stepped on the slopes after the start signal. A period of 15 s was recorded starting with the initial step. Each setup – standing on level ground (Level), on an upward slope (Up), and on a downward slope (Down) – was conducted three times. TF participants performed standing on a downward slope in total 6 times, three times with an enabled stance function of the prosthetic knee (locked joint in standing, referred as KSF on) and three times with a disabled stance function (free rotation joint in standing, referred as KSF off).

### Data processing

**Fig. 1:**
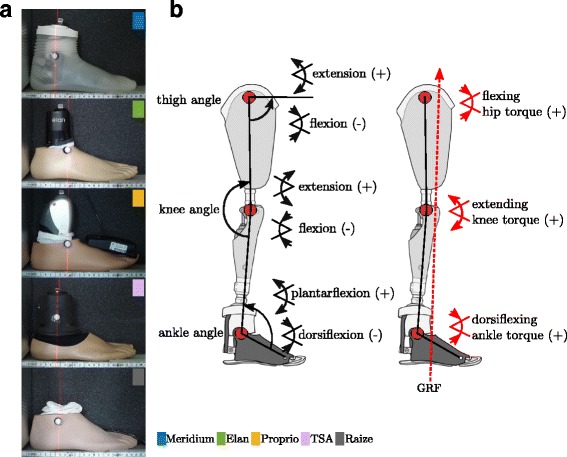
Definition of leg joint angles. **a** Modeled ankle joint position for the different MPF. The marker position corresponds to the mechanical joint axis of the foot. **b** Definition of used leg joint angles and sign convention

Kinematic and kinetic data were filtered (Woltring filter) and processed with Vicon Nexus™ 1.8/Workstation™ (Vicon Motion Systems, UK). With customized BodyBuilder scripts (Vicon Motion Systems, UK), the joint angles and torques of the ankle (angle between knee-ankle-toe markers), knee (angle between ankle-knee-Trochanter major markers) and thigh (angle between knee-Trochanter major - horizontal plane) in the sagittal plane were determined (Fig. 1). The joint angles were normalized to standing on level ground (Δα_joint,situation_ = α_joint,situation_–α_joint,level_). Joint torques are the sagittal moments acting at the joints due to the ground reaction force.

From each 15 s trial, a time period of one second was chosen when subjects stood quietly (usually at the end, e.g. from second 13 until second 14) for calculating the average force, angle and torque values. Afterwards the mean values for all variables were calculated for each situation (Level, Up, Down) and each group (TT, TF, non-Amp). Standard deviations (SD), maximum and minimum were determined for each group as well.

For the biomechanical parameters (Tables 4, 5 and 6), Mann-Whitney-U tests were performed to find statistically significant differences compared to the reference group (nonparametric test due to non-normal distribution, different group size, and large variation in variances). To account for multiple comparisons, a Bonferroni correction was used to adjust confidence intervals.

**Table 4:** Mean vertical ground reaction forces for the different situations and feet

		Prosthetic TT	Prosthetic TF	non-Amp
Level	Everyday Feet	49.8 ± 2.8	47.6 ± 6.1	50.0 ± 2.2
	Meridium	52.8 ± 2.8	46.5 ± 3.6	
	Elan	49.8 ± 3.2	47.0 ± 2.5	
	Proprio	48.7 ± 4.6	47.1 ± 4.1	
	TSA	52.4 ± 2.7	48.7 ± 3.1	
	Raize	51.5 ± 4.4		
Down (KSF on/off)	Everyday Feet	**38.5 ± 3.0**	48.9 ± 1.4 / **31.5 ± 5.7**	50.0 ± 2.8
	Meridium	51.3 ± 4.1	45.4 ± 3.2 / 44.6 ± 3.3	
	Elan	**42.7 ± 6.2**	48.6 ± 3.8 / **32.8 ± 16.3**	
	Proprio	**35.3 ± 3.8**	46.7 ± 1.9 / **33.8 ± 5.3**	
	TSA	45.6 ± 5.2	51.1 ± 0.7 / 48.2 ± 3.4	
	Raize	49.8 ± 4.4		
Up	Everyday Feet	48.4 ± 7.1	**41.1 ± 4.3**	50.0 ± 3.3
	Meridium	**55.0 ± 2.2**	47.4 ± 3.3	
	Elan	46.3 ± 5.7	**43.2 ± 4.9**	
	Proprio	**40.2 ± 2.8**	**40.4 ± 3.6**	
	TSA	50.6 ± 3.7	**42.4 ± 5.0**	
	Raize	45.7 ± 4.7		

Mean vGRF in %bw ± SD shown. Significant differences to the reference group (non-Amp) marked bold (*p* < 0.0083 for TT and *p* < 0.01 for TF). Note, the weight difference between sound and prosthetic leg is not considered in the statistical comparison

**Table 5:** Mean leg joint angles for the different situations and feet

			Prosthetic TT	Prosthetic TF	Sound TT	Sound TF	non-Amp
Δ ankle angle	Down (KSF on/off)	Everyday Feet	**4.9 ± 3.0**	3.9 ± 5.6 / 3.8 ± 7.2	9.6 ± 6.2	4.6 ± 5.4 / **3.9 ± 5.4**	10.1 ± 1.2
		Meridium	8.5 ± 1.0	9.5 ± 1.2 / 9.7 ± 1.3	9.2 ± 1.1	10.7 ± 0.7 / 10.7 ± 0.7	
		Elan	7.9 ± 3.0	**3.7 ± 4.0** / 5.2 ± 4.6	8.5 ± 1.9	5.6 ± 4.3 / 9.7 ± 5.0	
		Proprio	**2.7 ± 1.9**	**−0.6 ± 2.8** / **1.2 ± 3.6**	8.4 ± 3.7	**4.1 ± 6.2** / 8.0 ± 4.7	
		TSA	11.7 ± 2.8	6.1 ± 3.6 / 9.2 ± 2.8	11.7 ± 1.8	6.3 ± 4.4 / 9.0 ± 5.0	
		Raize	12.4 ± 2.2		11.5 ± 1.4		
	Up	Everyday Feet	**−1.9 ± 0.8**	−5.2 ± 3.9	−12.0 ± 4.7	−9.8 ± 5.6	−10.1 ± 1.3
		Meridium	−9.1 ± 0.7	−9.7 ± 1.9	−9.5 ± 1.7	−9.4 ± 1.4	
		Elan	**−3.3 ± 3.0**	−7.4 ± 1.5	−11.3 ± 1.1	−11.4 ± 1.8	
		Proprio	**−3.8 ± 1.6**	**−4.4 ± 1.1**	−12.6 ± 1.7	−11.0 ± 1.7	
		TSA	−7.5 ± 5.1	**−0.8 ± 0.7**	−10.6 ± 2.2	−12.6 ± 3.2	
		Raize	−4.4 ± 4.6		−12.9 ± 4.3		
Δ knee angle	Down (KSF on/off)	Everyday Feet	**−8.9 ± 7.9**	**−15.2 ± 8.1** / -15.5 ± 11.5	−4.3 ± 9.3	**−14.5 ± 12.3**/ -11.0 ± 12.1	−0.9 ± 2.1
		Meridium	−2.2 ± 2.5	−1.8 ± 2.1 / -0.5 ± 1.3	−0.3 ± 1.7	0.3 ± 1.1 / 0.4 ± 1.1	
		Elan	**−7.9 ± 2.5**	**−15.0 ± 5.7** / -9.9 ± 11.0	−1.8 ± 4.0	**−14.6 ± 5.7** / -4.0 ± 6.0	
		Proprio	**−13.2 ± 6.9**	**−22.2 ± 8.7** / -14.7 ± 15.7	−4.5 ± 6.8	**−17.4 ± 10.6** / -7.3 ± 9.9	
		TSA	−1.8 ± 2.0	**−8.1 ± 3.7** / -2.9 ± 4.2	−0.4 ± 2.7	**−13.7 ± 8.7** / -6.5 ± 8.8	
		Raize	−1.7 ± 2.6		0.4 ± 1.6		
	Up	Everyday Feet	2.2 ± 14.6	**0.3 ± 0.4**	−4.1 ± 6.8	−2.0 ± 4.2	−1.7 ± 1.8
		Meridium	0.5 ± 1.2	0.4 ± 0.8	−1.9 ± 1.4	−1.9 ± 2.3	
		Elan	**9.4 ± 2.9**	**0.8 ± 0.3**	−0.7 ± 0.7	−0.2 ± 2.0	
		Proprio	0.5 ± 15.4	**0.8 ± 0.6**	−3.2 ± 3.7	0.5 ± 0.9	
		TSA	4.3 ± 10.5	**0.5 ± 0.3**	−1.4 ± 1.5	−0.8 ± 2.7	
		Raize	3.9 ± 12.1		−3.3 ± 7.9		
Δ thigh angle	Down (KSF on/off)	Everyday Feet	**−7.9 ± 3.5**	**−9.8 ± 3.1** / **-10.9 ± 3.6**	−5.2 ± 3.1	**−10.2 ± 5.0** / **-8.1 ± 4.6**	−1.2 ± 2.2
		Meridium	−1.2 ± 1.5	−2.2 ± 1.6 / -1.1 ± 0.8	−0.2 ± 0.7	−0.7 ± 1.6 / -0.5 ± 0.8	
		Elan	**−6.4 ± 1.7**	**−9.8 ± 1.5** / **-8.5 ± 1.7**	−2.1 ± 1.8	**−10.0 ± 2.2** / **-4.8 ± 0.9**	
		Proprio	**−8.7 ± 2.1**	**−13.6 ± 5.1** / **-10.5 ± 7.5**	−4.4 ± 3.2	**−11.6 ± 4.9** / **-6.9 ± 4.5**	
		TSA	−6.4 ± 3.0	**−6.7 ± 1.8** / -4.4 ± 1.8	−3.2 ± 0.9	**−10.2 ± 5.1** / **-6.6 ± 5.0**	
		Raize	−4.7 ± 2.9		−2.6 ± 0.7		
	Up	Everyday Feet	0.9 ± 5.5	2.3 ± 2.1	−1.7 ± 1.8	1.7 ± 0.9	−0.4 ± 1.4
		Meridium	−0.3 ± 0.6	0.0 ± 1.6	−1.4 ± 0.8	−1.1 ± 1.3	
		Elan	**3.4 ± 1.5**	**2.5 ± 1.3**	−0.4 ± 1.2	**2.4 ± 1.7**	
		Proprio	2.3 ± 5.2	**1.7 ± 0.8**	−0.7 ± 2.5	**2.7 ± 1.6**	
		TSA	1.7 ± 4.9	2.1 ± 1.4	−0.7 ± 0.9	2.0 ± 1.1	
		Raize	1.0 ± 3.7		−1.4 ± 2.1		

Mean leg joint angles in ° ± SD shown. Significant differences to the reference group (non-Amp) marked bold (p < 0.0083 for TT and p < 0.01 for TF). Values are differences to level standing, i.e. ankle angle: positive – more plantar flexed, negative – more dorsiflexed; knee and hip angle: positive – more extended, negative – more flexed

**Table 6:** Mean leg joint moments for the different situations and feet

			Prosthetic TT	Prosthetic TF	Sound TT	Sound TF	non-Amp
Ankle torque	Level	Everyday Feet	0.38 ± 0.11	0.40 ± 0.09	0.36 ± 0.06	0.33 ± 0.05	0.26 ± 0.08
		Meridium	0.41 ± 0.09	0.33 ± 0.13	0.35 ± 0.10	0.31 ± 0.07	
		Elan	0.07 ± 0.09	0.07 ± 0.10	**0.34 ± 0.08**	0.31 ± 0.08	
		Proprio	0.22 ± 0.04	0.20 ± 0.03	0.35 ± 0.06	0.31 ± 0.07	
		TSA	0.36 ± 0.07	0.31 ± 0.04	0.32 ± 0.11	0.30 ± 0.06	
		Raize	0.32 ± 0.06		0.29 ± 0.13		
	Down (KSF on/off)	Everyday Feet	−0.02 ± 0.24	0.11 ± 0.24 / 0.15 ± 0.18	**0.43 ± 0.13**	**0.42 ± 0.14** / 0.44 ± 0.22	0.21 ± 0.08
		Meridium	0.38 ± 0.10	0.24 ± 0.05 / 0.24 ± 0.07	0.33 ± 0.08	0.29 ± 0.06 / 0.30 ± 0.10	
		Elan	**−0.10 ± 0.08**	**−0.05 ± 0.07** / **-0.11 ± 0.16**	**0.39 ± 0.08**	0.27 ± 0.12 / 0.37 ± 0.16	
		Proprio	**−0.10 ± 0.15**	0.09 ± 0.18 / 0.00 ± 0.20	**0.39 ± 0.03**	0.29 ± 0.09 / 0.35 ± 0.12	
		TSA	**0.04 ± 0.02**	**0.04 ± 0.04** / 0.06 ± 0.09	0.25 ± 0.13	0.32 ± 0.09 / 0.25 ± 0.04	
		Raize	**0.06 ± 0.03**		0.20 ± 0.14		
	Up	Everyday Feet	**0.62 ± 0.15**	**0.66 ± 0.07**	0.30 ± 0.13	0.51 ± 0.23	0.30 ± 0.06
		Meridium	0.32 ± 0.09	0.32 ± 0.04	0.36 ± 0.03	0.37 ± 0.11	
		Elan	**0.23 ± 0.01**	**0.48 ± 0.09**	0.26 ± 0.05	0.36 ± 0.07	
		Proprio	**0.46 ± 0.02**	**0.52 ± 0.04**	0.40 ± 0.10	0.45 ± 0.12	
		TSA	0.44 ± 0.16	**0.61 ± 0.08**	0.30 ± 0.12	0.39 ± 0.15	
		Raize	**0.52 ± 0.15**		0.23 ± 0.11		
knee torque	Level	Everyday Feet	0.12 ± 0.03	0.13 ± 0.02	0.15 ± 0.09	0.18 ± 0.06	0.14 ± 0.08
		Meridium	0.17 ± 0.05	0.11 ± 0.09	0.14 ± 0.10	0.15 ± 0.03	
		Elan	**−0.01 ± 0.06**	0.07 ± 0.03	0.15 ± 0.13	0.19 ± 0.10	
		Proprio	0.13 ± 0.02	0.13 ± 0.06	0.14 ± 0.09	0.13 ± 0.11	
		TSA	0.12 ± 0.06	0.14 ± 0.03	0.12 ± 0.08	0.14 ± 0.06	
		Raize	0.07 ± 0.06		0.11 ± 0.08		
	Down (KSF on/off)	Everyday Feet	**−0.16 ± 0.04**	**−0.21 ± 0.28** / -0.01 ± 0.03	0.12 ± 0.35	−0.02 ± 0.28 / -0.04 ± 0.33	0.10 ± 0.07
		Meridium	0.11 ± 0.03	0.06 ± 0.05 / 0.07 ± 0.06	0.11 ± 0.10	0.15 ± 0.01 / 0.16 ± 0.04	
		Elan	**−0.17 ± 0.04**	**−0.21 ± 0.05** / 0.00 ± 0.06	0.15 ± 0.16	**−0.07 ± 0.11** / 0.12 ± 0.17	
		Proprio	**−0.16 ± 0.06**	**−0.24 ± 0.03** / -0.01 ± 0.09	0.05 ± 0.25	−0.19 ± 0.26 / -0.04 ± 0.38	
		TSA	0.04 ± 0.07	**−0.09 ± 0.09** / 0.04 ± 0.02	0.08 ± 0.11	−0.04 ± 0.20 / -0.01 ± 0.16	
		Raize	**−0.03 ± 0.07**		0.07 ± 0.08		
	Up	Everyday Feet	0.34 ± 0.28	**0.29 ± 0.07**	0.07 ± 0.06	0.24 ± 0.14	0.14 ± 0.07
		Meridium	0.14 ± 0.01	0.11 ± 0.08	0.13 ± 0.10	0.21 ± 0.06	
		Elan	**0.26 ± 0.04**	**0.30 ± 0.06**	0.14 ± 0.11	0.24 ± 0.07	
		Proprio	**0.38 ± 0.06**	**0.31 ± 0.09**	0.12 ± 0.07	0.25 ± 0.08	
		TSA	0.21 ± 0.15	**0.31 ± 0.04**	0.10 ± 0.12	0.19 ± 0.10	
		Raize	0.29 ± 0.14		0.04 ± 0.11		
hip torque	Level	Everyday Feet	−0.02 ± 0.07	0.00 ± 0.08	−0.10 ± 0.05	0.03 ± 0.08	−0.06 ± 0.05
		Meridium	−0.02 ± 0.07	−0.02 ± 0.09	−0.07 ± 0.05	−0.06 ± 0.01	
		Elan	−0.01 ± 0.12	0.01 ± 0.06	−0.12 ± 0.10	0.07 ± 0.12	
		Proprio	**0.07 ± 0.06**	0.00 ± 0.05	−0.08 ± 0.08	−0.01 ± 0.10	
		TSA	0.00 ± 0.08	0.02 ± 0.06	−0.08 ± 0.10	0.00 ± 0.13	
		Raize	0.04 ± 0.08		−0.12 ± 0.09		
	Down (KSF on/off)	Everyday Feet	0.01 ± 0.08	0.09 ± 0.15 / **0.26 ± 0.12**	−0.08 ± 0.02	0.03 ± 0.08 / -0.04 ± 0.07	−0.06 ± 0.06
		Meridium	−0.05 ± 0.11	0.01 ± 0.10 / 0.00 ± 0.13	−0.09 ± 0.07	−0.06 ± 0.01 / -0.06 ± 0.03	
		Elan	0.04 ± 0.09	**0.10 ± 0.12** / **0.22 ± 0.15**	−0.13 ± 0.07	0.07 ± 0.12 / 0.05 ± 0.17	
		Proprio	**0.09 ± 0.04**	0.13 ± 0.18 / **0.24 ± 0.10**	−0.11 ± 0.10	−0.01 ± 0.10 / -0.10 ± 0.12	
		TSA	**0.14 ± 0.10**	0.09 ± 0.12 / **0.13 ± 0.04**	−0.08 ± 0.09	0.00 ± 0.13 / -0.06 ± 0.10	
		Raize	**0.12 ± 0.05**		−0.11 ± 0.12		
	Up	Everyday Feet	0.12 ± 0.24	0.03 ± 0.08	−0.08 ± 0.07	**0.05 ± 0.05**	−0.04 ± 0.06
		Meridium	−0.02 ± 0.10	−0.02 ± 0.17	−0.06 ± 0.05	0.04 ± 0.08	
		Elan	0.08 ± 0.14	0.05 ± 0.09	−0.05 ± 0.06	0.07 ± 0.15	
		Proprio	0.10 ± 0.14	0.05 ± 0.07	−0.03 ± 0.10	0.02 ± 0.09	
		TSA	0.05 ± 0.09	0.04 ± 0.07	−0.06 ± 0.13	−0.03 ± 0.11	
		Raize	**0.15 ± 0.14**		−0.07 ± 0.15		

Mean leg joint moments in Nm/kg ± SD shown. Significant differences to the reference group (non-Amp) marked bold (p < 0.0083 for TT and p < 0.01 for TF). Ankle torques: positive – dorsiflexing, negative – plantar flexing; knee torques: positive – extending, negative – flexing; hip torques: positive – flexing, negative – extending. Note, the torques acting on the prosthetic ankle joint are only conditionally comparable between the feet and to non-amputees, since the (posterior-anterior) position of the mechanical ankle joint depends on the foot

## Results

### Vertical ground reaction forces

**Fig. 2:**
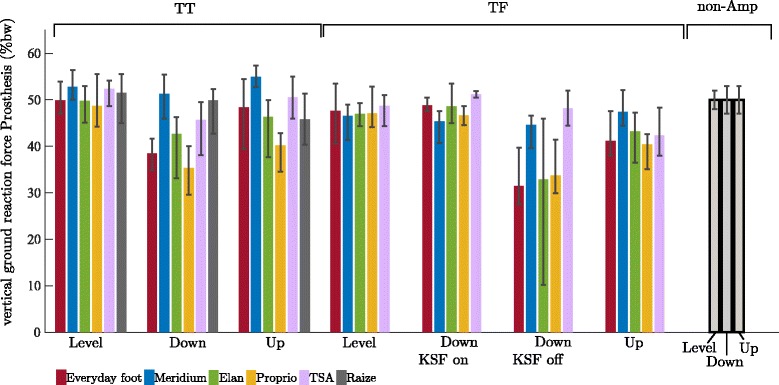
Vertical ground reaction forces for TT and TF amputees sorted by foot and situation. The investigated feet are (from left to right): Everyday foot (red), Meridium (blue), Elan (green), Proprio (orange), TSA (purple), and Raize (grey only TT). The error bars specify maximum and minimum within the group. For comparison, the mean and SD values of the non-amputee group (black-framed) are given, too. Note, that the colored bars give the vGRF of the prosthetic side (mean values in % body weight) while the vGRF of the sound side is the remaining amount (vGRF(sound) = 100 – vGRF(prosthetic) in %)

For standing on level ground, we found almost symmetrical vertical ground reaction forces (vGRF in % body weight: %bw) between prosthetic and sound sides for TTs (from 53%bw to 49%bw) and a slightly but not significantly increased vGRF for the sound side of TFs (from 51%bw to 54%bw) for all feet. The reference group showed a symmetrical vGRF distribution (Fig. 2 and Table 4).

For standing on an upward slope of 10°, the vGRF distribution between the prosthetic and sound sides changed depending on the foot used (Fig. 2 and Table 4). Decreased, almost unaffected, and increased vGRFs were found for TTs (from 55%bw for Meridium to 40%bw for Proprio; sign. effects could not be linked to features). For all feet, the TF subjects loaded the prosthetic side less ranging from 47%bw for Meridium to 40%bw for Proprio and Everyday feet (sign. differences for feet without an auto-adaptive dorsiflexion stop).

On a −10° downward slope, TT subjects showed decreased (e.g., down to 35%bw for Proprio; sign. differences for feet without sufficient ROM), or almost unaffected vGRFs (Fig. 2 and Table 4). For TF subjects, we investigated standing on a downward slope of −10° under two conditions: first, with an enabled knee stance function (KSF on) in the prosthetic knee used and, second, with disabled stance function in the knee (KSF off). Note that the stance function of the prosthetic knee used (Genium) was enabled when standing on level ground and upward slopes. With KSF on, the differences between feet and to non-amputees when standing on level ground were small and not significant, ranging from 45%bw for Meridium to 51%bw for TSA (Fig. 2 and Table 4). With KSF off, however, the subjects loaded the prosthesis less (sign. differences for feet without sufficient ROM), ranging from 31%bw for Everyday feet to 48%bw for TSA.

### Joint torques

**Fig. 3:**
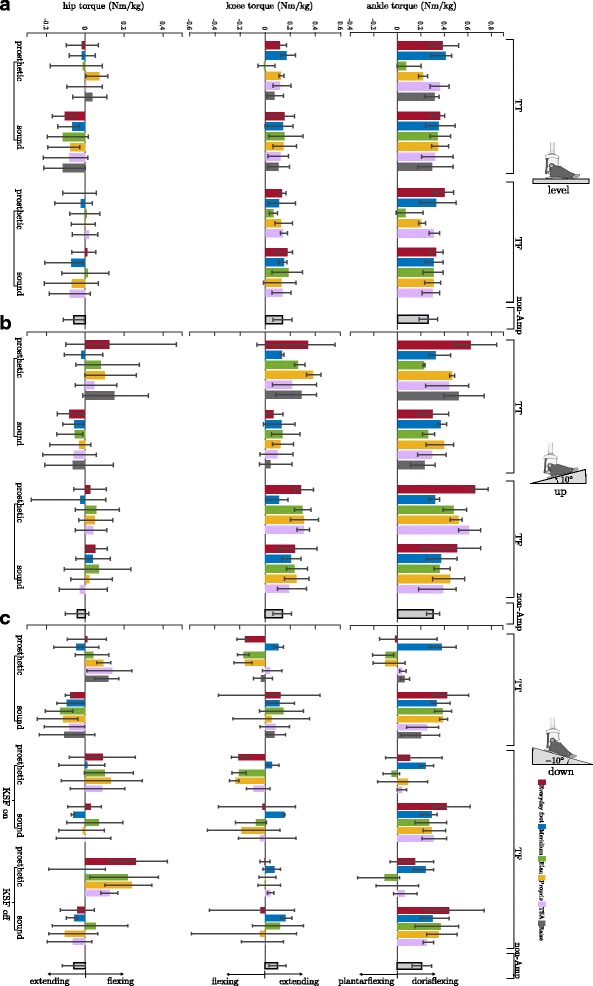
Leg joint torques for standing on level ground, on an upward slope, and on a downward slope. **a** level ground, (**b**) on an upward slope of 10°, and (**c**) on an downward slope of −10° for TT and TF amputees. Sorted by foot and situation. Feet are (from left to right): Everyday foot (red), Meridium (blue), Elan (green), Proprio (orange), TSA (purple), and Raize (grey only TT). The error bars specify maximum and minimum within the group for the amputees. Mean and SD values of the non-amputee group (black-framed) are given, too. Mean values in Nm/kg. Note, for amputees the knee (TT) and hip (TT & TF) torques on the prosthetic side and all of the sound side are the crucial ones (not the torques acting on the prosthesis). From an engineering point of view the torques acting on the prosthesis are also interesting

The joint torques are reported in Table 6 and Fig. 3. The highest torques were acting on the ankle joints. On the sound ankle joint, only dorsiflexing torques were observed. On the prosthetic side, both dorsi- and plantarflexing torques were observed (foot- and situation dependent). In the reference group only dorsiflexing torques were observed for all situations.

For the knee and hip joints foot- and situation dependent torques were observed as well. Torques were found to be acting extending, flexing, and fluctuating around zero. In the reference group, for these joints only extending torques were found (Fig. 3 and Table 6).

In summary, no significant differences were found for the residual limb joints in feet with additional ROM and an auto-adaptive dorsiflexion stop whereas the other foot concepts cause many significant differences (Table 5).

### Ankle angle adaptations

**Fig. 4:**
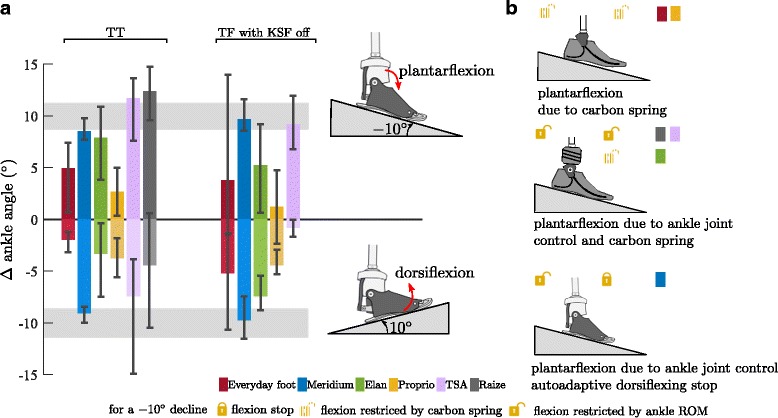
Ankle angle adaptations in the prosthetic feet to upward and downward slopes. **a** Ankle angle adaptation in the prosthetic feet compared to level standing for TT and TF (KSF off) subjects for standing on an upward and downward slope of 10°. Feet are (from left to right): Everyday foot (red), Meridium (blue), Elan (green), Proprio (orange), TSA (purple), and Raize (grey only TT). The error bars specify maximum and minimum within the group for the amputees. Range of adaptation (±SD) found in the non-amputee group is shaded in gray. Note that due to compensatory strategies in knee and hip the ROM of the MPF might not be exploited. **b** Mechanisms causing plantarflexion while standing on a −10° downward slope

**Fig. 5:**
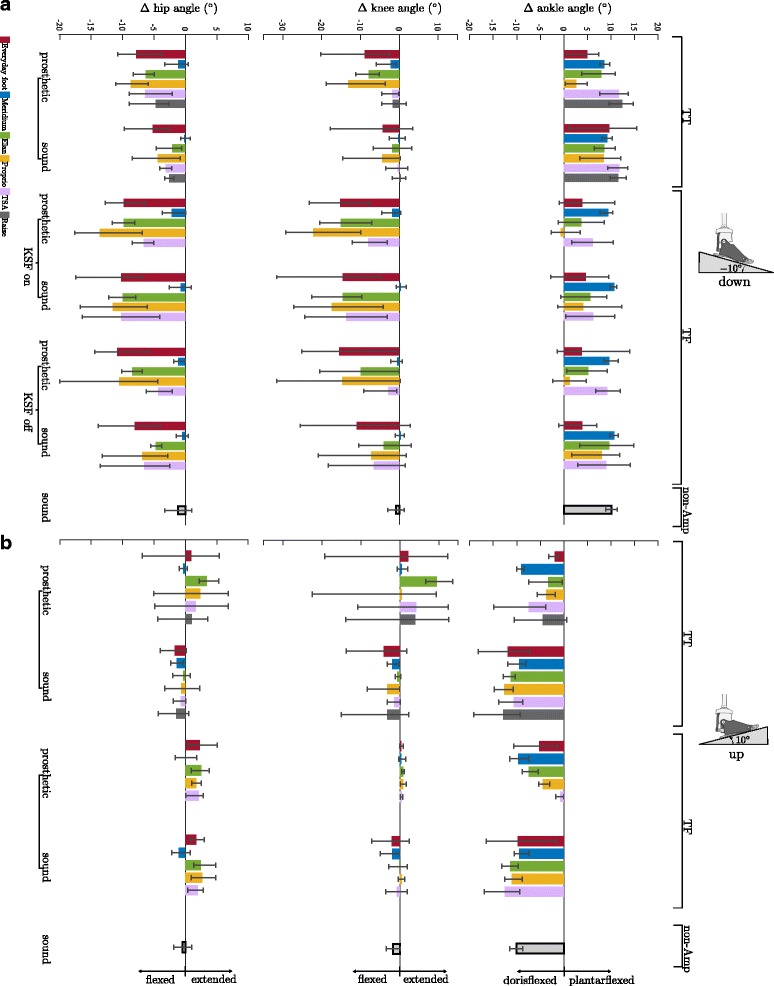
Leg joint angles for standing on a downward and upward slope. **a** downward slope of −10° and (**b**) upward slope of 10° for TT and TF amputees sorted by foot and side. Feet are (from left to right): Everyday foot (red), Meridium (blue), Elan (green), Proprio (orange), TSA (purple), and Raize (grey only TT). Values shown are differences to level standing. The error bars specify maximum and minimum within the group for the amputees. The mean and SD values of the non-amputee group (black-framed) are given, too

For standing on a downward slope, adaptations in the ankle angle of the prostheses (i.e. Δα, see Data processing) were observed, ranging from −0.6° (Proprio) to 12.4° (Raize) (Fig. 4 & 5A and Table 5). For the corresponding sound side, adaptations were observed, ranging from 3.9° (Everyday foot) to 11.7° (TSA). Note that some subjects showed a dorsiflexed foot position (TF for Everyday foot, Elan, and Proprio).

For standing on an upward slope, the feet became more dorsiflexed. On the prosthetic side, adaptations ranged from −0.8° (TSA) to −9.7° (Meridium) (Fig. 4 & 5B and Table 5).

The reference group showed adaptations of 10 ± 1° for Down and −10 ± 1° for Up. A full adaptation of the foot to the inclination results in adaptations of the ankle angle of 10° for Up and −10° for Down if the posture proximal to the ankle joint is not altered compared to standing on level ground. It also indicates that most of the compensation is achieved in the ankle joint.

### Knee and thigh angles adaptations

Situation-dependent adaptations were found in knee and thigh angles as well. In general, these adaptations are small for TFs while standing on an upward slope (both legs, variations about 2°) as well as for all situations when amputees were equipped with a Meridium (Fig. 5 and Table 5). For the other feet and situations, the adaptations and inter-individual variations were larger. Here the hip and knee joints showed similar adaptations in direction and magnitude (Fig. 5 and Table 5).

In summary, only one significant difference was found for the residual limb joints in feet with additional ROM and none for feet with additional ROM and an auto-adaptive dorsiflexion stop as compared with feet without, with limited, or unused additional ROM (Table 5).

## Discussion

It is generally known that prosthetic feet influence the walking performance in lower leg amputees. However, to what extent current foot concepts support physiological standing on uneven ground was not yet clear. By investigating lower limb amputees standing on slopes of different inclinations, it has now been demonstrated that the functionality and adaptability of the prosthetic foot used are the keys for a more natural posture and improved symmetric loading of the legs. Two key features could be identified: an auto-adaptive dorsiflexion stop and sufficient range of motion (ROM) for adaptation.

### Standing on level ground is not challenging

Standing on an even ground does not appear to be challenging since the prosthetic alignment is optimized for standing and walking on level ground. For such a situation almost symmetrical vGRF can be observed for the different feet. The subjects in our study loaded about 51%bw for TT and about 47%bw for TF on the prostheses. Especially for TTs, this is similar to non-amputees. The slightly reduced load on the prostheses for TFs has been previously reported in literature (e.g. [28–30], for a review see [25]). An estimate revealed that 47%bw on the prosthesis and 53% on the sound side is close to a symmetrical loading in non-amputees due to loss of leg weight (80 kg non-amp, 6 kg TF prosthesis, 6%bw loss in total, anthropometric human data of [31]). The kinetic parameters of the ankle and knee on the sound side are similar among the different feet and comparable to non-amputees (mean of TT and TF within the SD of non-amp as shown in Fig. 3). Furthermore, we found the same effects for the hip torques of the sound side and for the knee torques of TTs prosthetic side with some exceptions (Fig. 3 and Table 6). The torques acting on the prosthetic ankle joint are not directly comparable between the feet and to non-amputees, since the (posterior-anterior) position of the mechanical ankle joint depends on the foot design (Fig. 1) and, therefore, influences the lever arm. However, one MPF showed noticeably small dorsiflexing moments, which are related to its functionality (Elan, see also below). This foot concept does not provide an auto-adaptive dorsiflexion stop. Instead, the user balances on a free ankle joint (hydraulically damped, ROM limited to 6° plantarflexion and 3° dorsiflexion) or might utilize the end position of dorsiflexion ROM (3°) as stop with effects on the posture.

### Implemented features explain behavior on slopes

The influence of the different features embedded in the MPF become evident when analyzing standing on slopes with different inclinations. The vGRF distribution between prosthetic and sound side is affected. The biggest differences between feet and in comparison with level standing can be found for standing on a downward slope. In this situation, feet that are capable of fully adjusting the ankle joint in plantarflexion to the −10° inclination show only minor adaptations in the vGRF (Meridium, TSA, Raize, see Fig. 3 and Table 4 TT and TF KSF off; for features see Tables 2 and 3). Similar findings have been reported for a prototype foot which is able to adapt to inclinations [22]. For feet which are not or only partly (due to limited ROM) able to adapt to the inclination, a major weight shift of up to 20%bw towards the sound side was found resulting in a compensatory posture (Everyday feet, Elan, Proprio - ROM not used due to swing phase adaptation; Fig. 2 & 5 and Table 5). TF subjects equipped with a prosthetic knee joint that is able to lock in standing do not exhibit the weight shift to the sound side, irrespective of the foot used. However, there are clear adaptations in posture for the different feet (Fig. 5, Table 5). On the one hand, a normal posture similar to standing on level ground can be observed when using feet that adapt to the inclination; on the other hand, knee and hip joints can be flexed significantly for compensation when using feet that do not provide such a feature. For standing on an upward slope, the same conclusions regarding the vGRF distributions and the dorsiflexion adaptability can be drawn for TFs with a less pronounced effect size while for TTs clear correlations could not be shown.

The torques acting on the leg joints in the sagittal plane are directly influenced by both, the vGRF distributions between the legs and the posture (joint angles). Consequently, the analysis of these torques reveals additional information about the functionality of the feet if compared to the analysis of vGRF distribution alone. For example, for TT downward standing, three of five MPF showed almost unaffected vGRF distributions, while only one of them applied an almost unaffected ankle torque compared to level standing. The unaffected vGRF can be attributed to the angular adaptability and the unchanged joint torque to an additional auto-adaptive dorsiflexion stop. It reveals that the ability of a foot to adapt in a plantarflexing manner alone does not necessarily enable the subject to stand with a natural posture.

Linked to the embedded features, major differences were not only found in the adaptation of the ankle torques to the situation but also in proximal joints. For the downward slope, knee flexing torques were found for feet that are not or only partly able to adapt to the decline (Elan, Proprio, Everyday feet). Torques fluctuated around zero (flexing/extending) for feet capable of plantarflexion more than 10° without a locking mechanism (TSA, Raize), and extending torques were observed if the dorsiflexion was combined with an auto-adaptive dorsiflexion stop (Meridium), (Fig. 3 and Table 6). Hip torques have to counterbalance lower joint torques. Therefore, hip torques also revealed differences compared to non-amputees when the knee torques were affected.

From the observations in our subjects, it can be deduced that the ability of the foot to lock once fully adapted to the inclination, i.e. to prevent movement in dorsal direction after adaptation, is the key for a nearly physiological posture while standing on slopes. For lower limb amputees, such a posture includes an almost equal distribution of vGRF between prosthetic and sound side. A physiologic posture would also require joint angles and joint torques that are comparable to non-amputees. Only one of the investigated feet, the Meridium, was able to fulfill these criteria.

### Influence of prosthetic knee functionality on posture

In this study, all TF amputees were equipped with microprocessor-controlled knee joints (Genium) which are able to lock the knee joint in quiet standing [5]. For standing on a downward slope, the subjects were investigated with this “stance function” in both, enabled and disabled mode. As mentioned above, the subjects showed only minor adaptations in vGRF distribution with an enabled mode, but feet-dependent adaptations were observed in the disabled mode. For the disabled mode, the adaptations in vGRF distribution were similar to the findings for TT amputees and knee torques were balanced about zero reflecting that flexing torques have to be counterbalanced by the residual limb. Under these conditions, the hip extensors have to increase the effort to stabilize standing (even with a decreased vGRF) resulting in increased flexing hip torques (Fig. 3 and Table 6, except for Meridium). Consequently, it appears that for knee joints without stance function, a fully adapting foot concept with a dorsiflexion stop appears to be beneficial for standing on slopes.

### Individual posture strategies

**Fig. 6:**
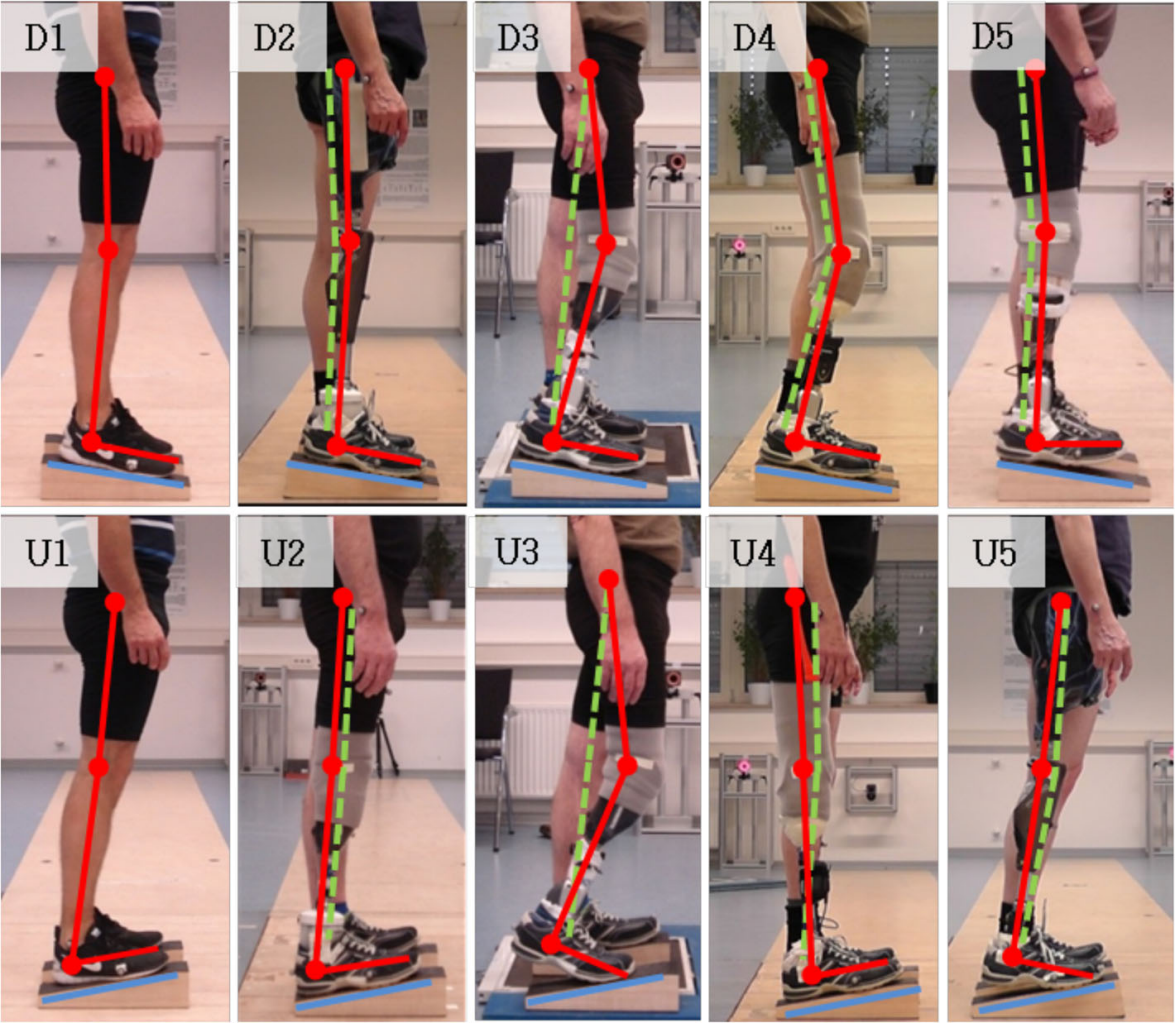
Individual postural strategies in standing on inclinations. Strategies used while standing on an upward slope of 10° (lower array) and on a downward slope (upper array). The individual posture is highlighted with red (prosthetic side) and dashed-green (sound side) bars for amputees (D2-D5 and U2-U5). The pictures in the first column (D1 and U1) show the posture of a non-amputee. It is almost identical with the posture of amputees equipped with a prosthetic foot that adapt fully to the inclinations and apply a dorisflexing stop (D2 and U2). The amputees use compensational postures if equipped with feet not adapting to the incline (D3-D5 and U3-U5). Note that the different individual compensation strategies shown in D3-D5 are the outcome of the same MPF

The observed differences in joint angles and torques between different prosthetic foot designs and the large inter-individual differences are outcomes of individual strategies that amputees develop over years of using prosthetic feet that are not fully adaptable. Four individual postural strategies for downward and upward standing are shown in Fig. 6, which are different to quiet standing of non-amputees. In this classification, it has been distinguished whether the sole of the foot is parallel to the surface of the slope and if the joint angle of the knee is bend or flexed. Furthermore, depending on the posture of the leg, the upper body is tilted to ensure static stability by shifting the center of mass in anterior-posterior direction (Fig. 6 U4). If the prosthetic foot is not fully capable of a 10° plantarflexion while standing on the downward slope, some subjects flex their knees to achieve a full foot contact (Fig. 6 D3-D4). Using such a posture, the flexing torque acting on the knee has to be compensated by the knee extensor muscles which is more exhausting than an upright posture. A similar adaptation has previously been reported [22]. Another possible strategy is to balance on the heel (Fig. 6 U5). This is also exhausting for the knee extensor muscles since the center of pressure is located posteriorly thus flexing the knee. Furthermore, this is more demanding concerning stability due to the smaller base of support [32]. For standing on an upward slope, one strategy with a flexed knee was observed (Fig. 3 U3). The other subjects extended the knee joints and used their body weight to dorsiflex the carbon spring (Fig. 6 U4-U5, see [33, 34] for similar hip-compensation strategies).

The postural strategies of the amputees while standing were freely chosen by the individuals (see methods). As discussed above, this leads to large inter-individual variations in the investigated parameters. Therefore, the limitations of the feet (ROM, functionality) might not be exploited in all subjects. Despite the different individual strategies of the amputees while wearing their everyday feet, the subjects adopted a more physiological posture after being equipped with a prosthetic foot capable of full adaptation to an incline and decline combined with an auto-adaptive dorsiflexion stop (Figs. 6 D1-D2 and U1-U2). Another effect of the improved posture is that the inter-individual variations as a measure of the different compensation strategies are significantly reduced to values of non-amputees.

### Limitations of the study

For the present study, we used different commercially available prosthetic feet. The Elan foot (Blatchford) had no stance function and a hydraulically damped rotating joint while standing (Table 3, [16]). Blatchford developed a new version of the Elan foot that is commercially available with a controlled increased damping while standing.

We investigated four TT and four TF amputees which exhibited large inter-individual differences in the studied parameters. Therefore, the statistical power is limited. The activity levels of the subjects were K3 to K4, which indicated that all were physically active in daily life. A direct transfer of these findings to lower mobility grades (K2) could be assumed, but has to be confirmed. Actually, amputees with lower mobility grades might benefit even more from prosthetic feet capable of a full adaptation to uneven ground and an auto-adaptive dorsiflexion stop than highly active people. It might prevent falls due to an increased stability that counteracts balance problems and reducing necessary muscle strength as a result of a more natural posture.

**Fig. 7:**
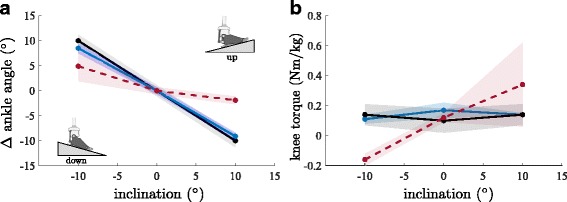
Interpolation to a range of inclinations. **a** Ankle angle adaptations in the prosthetic feet compared to level standing and (**b**) knee torques for TT. Feet are: Everyday foot (red), Meridium (blue), non-Amp (black). The shaded areas are the corresponding SD

This study only investigated standing on inclines and declines of 10° in comparison to level ground so that statements about lesser degrees can only be interpolated based on the present results (Fig. 7). Furthermore, a conclusion if features that help amputees in standing show disadvantages in other movement-specific tasks cannot be drawn. Nonetheless, the participants did not report disadvantages that could be linked to special features. In general, the higher functionality of the MPF comes at the cost of increased prosthetic foot weight, energy loss due to hydraulic damping (if implemented) and the need for regular charging.

## Conclusions

A prosthetic foot that combines both key features - an auto-adaptive dorsiflexion stop and sufficient ROM to completely adapt to inclinations - enables lower limb amputees to stand on slopes in an almost natural manner. The biomechanical parameters indicate that this concept is superior to conventional passive feet or feet which provide only one key design feature such as a sufficient ROM. Finally, the results indicate that both, TT and TF amputees, benefit from such a foot.

## Abbreviations

Bw: Body weight
Down: standing on a downward slope
KSF: prosthetic knee stance function
Level: standing on level ground
MPF: Microprocessor-controlled prosthetic feet
non-Amp: non-amputees of reference group
ROM: range of motion
TF: Transfemoral amputee
TT: Transtibial amputee
Up: standing on an upward slope
vGRF: vertical ground reaction force

## References

[1] Segal AD, Orendurff MS, Klute GK (2006). Kinematic and kinetic comparisons of transfemoral amputee gait using C-leg and Mauch SNS prosthetic knees. J Rehabil Res Dev.

[2] Kaufman KR, Levine JA, Brey RH (2007). Gait and balance of transfemoral amputees using passive mechanical and microprocessor-controlled prosthetic knees. Gait Posture.

[3] Schmalz T, Blumentritt S, Marx B (2007). Biomechanical analysis of stair ambulation in lower limb amputees. Gait Posture.

[4] Bellmann M, Schmalz T, Blumentritt S (2010). Comparative biomechanical analysis of current microprocessor-controlled prosthetic knee joints. Arch Phys Med Rehabil.

[5] Bellmann M, Schmalz T, Ludwigs E, Blumentritt S (2012). Immediate effects of a new microprocessor-controlled prosthetic knee joint: a comparative biomechanical evaluation. Arch Phys Med Rehabil.

[6] Highsmith MJ, Kahle JT, Lura DJ, Lewandowski AL, Quillen WS, Kim SH (2014). Stair ascent and ramp gait training with the genium knee. Technol Innov.

[7] Kahle JT (2008). Comparison of nonmicroprocessor knee mechanism versus C-leg on prosthesis evaluation questionnaire, stumbles, falls, walking tests, stair descent, and knee preference. J Rehabil Res Dev.

[8] Hafner BJ, Smith DG (2009). Differences in function and safety between Medicare functional classification Level-2 and -3 transfemoral amputees and influence of prosthetic knee joint contro. J Rehabil Res Dev.

[9] Blumentritt S, Schmalz T, Jarasch R (2009). The safety of C-leg: biomechanical tests. J Prosthet Orthot.

[10] Highsmith MJ, Kahle JT, Shepard NT, Kaufman KR (2013). The effect of the C-leg knee prosthesis on the sensory dependency and falls during sensory organization testing. Technol Innov.

[11] Highsmith MJ, Kahle JT, Wernke MM (2016). Effects of the genium knee syste, on the functional level, strair ambulation, perceptive and economic outcomes in transfemoral amputees. Technol Innov.

[12] Hahn A, Lang M (2015). Effects of mobility grade, age, and etiology on functional benefit and safety of subjects evaluated in more than 1200 C-leg trial fittings in Germany. J Prosthet Orthot.

[13] Fey NP, Klute GK, Neptune RR (2011). The influence of energy storage and return foot stiffness on walking mechanics and muscle activity in below-knee amputees. Clin Biomech.

[14] Su P, Gard SA, Lipschutz RD, Kuiken TA (2010). The effects of increased prosthetic ankle motions on the gait of persons with bilateral transtibial amputations. Am J Phys Med Rehabil.

[15] Huang TP (2015). Shorter KA, Adamczyk PG, Kuo AD. Mechanical and energetic consequences of reduced ankle plantar-flexion in human walking. J Exp Biol.

[16] Asha AR (2013). De, Munjal R, Kulkarni J, Buckley JG. Walking speed related joint kinetic alterations in trans-tibial amputees: impact of hydraulic 'ankle' damping. J Neuroeng Rehabil.

[17] Asha AR, De MR, Kulkarni J, Buckley JG (2014). Impact on the biomechanics of overground gait of using an 'Echelon' hydraulic ankle-foot device in unilateral trans-tibial and trans-femoral amputees. Clin Biomech.

[18] Caputo JM, Collins SH (2014). Prosthetic ankle push-off work reduces metabolic rate but not collision work in non-amputee walking. Sci Rep.

[19] Malcolm P, Quesada RE, Caputo JM, Collins SH (2015). The influence of push-off timing in a robotic ankle-foot prosthesis on the energetics and mechanics of walking. J Neuroeng Rehabil.

[20] Adamczyk PG, Kuo AD (2015). Mechanisms of gait asymmetry due to push-off deficiency in unilateral amputees. IEEE Trans Neural Syst Rehabil Eng.

[21] Herr HM, Grabowski AM (2012). Bionic ankle-foot prosthesis normalizes walking gait for persons with leg amputation. Proc R Soc B.

[22] Shultz AH, Lawson BE, Goldfarb M (2016). Variable cadence walking and ground adaptive standing with a powered ankle prosthesis. IEEE Trans Neural Syst Rehabil Eng.

[23] Grimmer M, Holgate M, Holgate R (2016). A powered prosthetic ankle joint for walking and running. Biomed Eng Online.

[24] Roaas A, Andersson GBJ (1982). Normal range of motion of the hip, knee and ankle joints in male subjects, 30–40 years of age. Acta Orthop Scand.

[25] PX K, Abu Osman NA, Wan Abas WAB (2014). Balance control in lower extremity amputees during quiet standing: a systematic review. Gait Posture.

[26] Orendurff MS, Raschke SU, Winder L, Moe D, Boone DA, Kobayashi T (2016). Functional level assessment of individuals with transtibial limb loss: evaluation in the clinical setting versus objective community ambulatory activity. J Rehab Ass Tech Eng.

[27] Nietert M (2008). The compromise pivot axis of the knee joint: studies of the kinematics of the human knee joint in regard to their approximation in prosthetics.

[28] Nederhand MJ, van Asseldonk EHF, van der Kooij H, Rietman HS (2012). Dynamic balance control (DBC) in lower leg amputee subjects; contribution of the regulatory activity of the prosthesis side. Clin Biomech.

[29] Nadollek H, Brauer S, Isles R (2002). Outcomes after trans-tibial amputation: the relationship between quiet stance ability, strength of hip abductor muscles and gait. Physiother Res Int.

[30] Mayer A, Tihanyi J, Bretz K, Csende Z, Bretz E, Horvath M (2011). Adaptation to altered balance conditions in unilateral amputees due to atherosclerosis: a randomized controlled study. BMC Musculoskelet Disord.

[31] Leva P (1996). De. Adjustments to Zatsiorsky-Seluyanov's segment inertia parameters. J Biomech.

[32] Hof AL, Gazendam MGJ, Sinke WE (2005). The condition for dynamic stability. J Biomech.

[33] Horak FB, Nashner LM (1986). Central programming of postural movements: adaptation to altered support-surface configurations. J Neurophysiol.

[34] Winter DA (1995). Human balance and posture control during standing and walking. Gait Posture.

